# Synergistic Reduction in Asphalt VOC Emissions by Hydrochloric Acid-Modified Zeolite and LDHs

**DOI:** 10.3390/ma17225664

**Published:** 2024-11-20

**Authors:** Haowei Zhao, Anqi Chen, Shaopeng Wu, Haiqin Xu, Huan Wang, Yang Lv

**Affiliations:** State Key Laboratory of Silicate Materials for Architectures, Wuhan University of Technology, Wuhan 430070, China; zhaohaowei@whut.edu.cn (H.Z.);

**Keywords:** hydrochloric acid-modified zeolite, LDHs, volatile organic compounds, adsorption, GC-MS, VOCs emission

## Abstract

Asphalt releases a large number of irritating fumes during construction and use, which is a serious emission pollution that not only damages the atmospheric environment but also produces highly toxic and carcinogenic volatile organic compounds (VOCs), posing a health risk to human beings. In this study, a compound-doped modified bitumen for reducing VOC emission was prepared by using zeolite as the main adsorbent material, modified by hydrochloric acid, and LDHs as a synergistic adsorbent material. By determining its basic and rheological properties, the results show that the compounding of LDHs and HCL-modified zeolite added to asphalt can improve the high-temperature performance of asphalt binder, but at the same time, the anti-fatigue property will be decreased. By GC-MS experimental analysis, a total of 72.2% fewer volatile organic compounds (VOCs) were released by the compound modified asphalt compound than by virgin asphalt, which resulted in a significant reduction in asphalt fume emissions. It shows that the asphalt VOC molecules are well adsorbed by the porous adsorption of LDHs and zeolite materials, and it is also found experimentally that they inhibit the emission of VOCs through the blocking and adsorption effects. This study provides a scientific basis for inhibiting the emission of VOCs during asphalt pavement construction.

## 1. Introduction

The expansion of the national road infrastructure has resulted in a significant increase in the total mileage of highways in China. At the beginning of 2024, the total mileage of highways in China reached 184,000 km, with the total mileage of highways reaching nearly 5,441,000 km. This places China at the forefront of global road construction, with the highest total mileage of any country in the world [1]. In recent years, about 200 billion RMB has been invested in road construction every year, and most of the road surfaces used in the construction of high-grade highways are made of asphalt, and the utilization rate of asphalt has become higher and higher than that of concrete, which is mainly attributed to the excellent performance of asphalt [2,3]. However, at the same time, a large amount of asphalt pavement will also produce corresponding harm. Asphalt is a complex, thick, semi-solid mixture of organic hydrocarbons and their derivatives with different molecular weights, different stability, and complex types [4,5]. Therefore, while we have to use asphalt, we must pay attention to this hazard. Both the previous energy saving and emission reduction and the green transportation concept of transportation powerhouse are always advocating the promotion of green building [6,7]. Increasing the promotion and application of green technology, promoting comprehensive green transformation of social and economic development, winning the battle against pollution, and realizing the goal of carbon peak and carbon neutrality has been the focus of China’s construction [8]. Therefore, to improve the smoke and irritating flavor produced during the construction and production of asphalt pavement, the preparation of a green asphalt material is an imperative trend, both for human health and environmental pollution [9].

VOCs are mainly derived from asphalt, petroleum, coal, or other petroleum derivatives, which cannot be oxidized deeply due to an insufficient supply of oxygen during production or construction, and the light components volatilize into the air in the form of aerosols after being heated [10,11]. Asphalt, a viscoelastic material, requires heating to a temperature of 160–180 °C when mixed. For modified asphalt, a widely used material in China, the mixing and construction temperature may be even higher, which results in the production of visible smoke during construction, a pungent odor, and impaired vision, creating challenges for construction operations [12,13,14]. The phenomenon of asphalt fumes is more prevalent in the context of urban road construction, factory construction, and tunnel construction [15]. Asphalt paving fumes accumulate on the construction surface, generating large amounts of smoke in narrow areas, causing inhalation hazards and reducing local oxygen levels, which can cause paver stalling and thus affect the service life of construction equipment [16,17]. In addition, asphalt fumes can pose a potential health risk to construction workers through respiratory inhalation and surface skin contact, adversely affecting the airways, lungs, liver, and kidneys of longtime workers and requiring medical attention in severe cases [18,19]. In the United States, more than 500,000 workers are exposed to asphalt fumes during operations, and studies have found that roofers have a higher risk of developing the same cancers than paving and road repair workers. Roofers have the same cancer risk compared to road paving and highway maintenance workers because they are more likely to cause inhalation hazards by burning melted asphalt, which cannot be easily controlled, and secondary hazards caused by VOCs that destroy the ozone layer and accelerate the greenhouse effect [20,21].

As society has developed, the issue of how to treat asphalt fumes has become a matter of great urgency. In recent years, it has been established that the majority of domestic and foreign technologies employed to mitigate the emission of asphalt fumes utilize adsorption methodologies [22,23]. The VOC inhibitors are mainly inorganic porous materials, and the range of choices is relatively small [24]. Bitumen is an organic mixture with a complex structure and composition, consisting of saturated hydrocarbons, aromatic hydrocarbons, resins, and asphaltenes [25,26]. However, common adsorbents are generally inorganic adsorbents such as zeolites and activated carbon [27,28]. The majority of studies have demonstrated that the optimal dosage of zeolite as a modifier for blending with asphalt is 3%. This dosage has been shown to result in superior asphalt properties [29,30]. At the same time, zeolite has an excellent adsorption effect compared to other inorganic adsorbents, and LDHs can better block the fumes that cannot be absorbed by zeolite in asphalt to prevent fume volatilization [31,32]. LDHs (Layered Double Hydroxides), collectively called layered bimetallic hydroxides, are hydroxides with a layered structure consisting of two or more metal cations and interlayer anions. The incorporation of hydrochloric acid-modified zeolite into SBS asphalt pairs will reduce the VOC emission of SBS asphalt, but at the same time, it will also reduce the comprehensive performance of SBS asphalt. Wang et al. used hydrochloric acid-modified zeolite as a VOCs inhibitor for 70# matrix asphalt. The results showed that the addition of zeolite reduced the total VOCs concentration by 0.43%, increased the softening point of asphalt by 5 °C, reduced the needle penetration by 2.2 mm, and reduced the ductility by 7 cm [33]. Chen et al. used alumina hydrotalcite as an asphalt modifier and investigated the anti-aging properties of modified asphalt. The results of the study showed that with the addition of alumina hydrotalcite, asphalt aging resistance increased by 21.4%, the carbonyl increase after aging modified asphalt is significantly higher than that of the carbonyl increase after aging modified asphalt TFOT, the sulfoxide group compared with the carbonyl change did not change significantly [34]. In this study, a mixture of LDHs and hydrochloric acid-modified zeolite was mixed into asphalt while ensuring a reasonable dosage [35,36]. The principle is due to the fact that the pores of the zeolite itself are clogged, and hydrochloric acid is known for its excellent modifying properties; therefore, the treatment of hydrochloric acid becomes extremely critical [37].

Asphalt low VOC emissions, which treat the symptoms as well as the root causes, this study through the study of asphalt smoke composition, inhibition methods, and smoke suppression mechanism, aims to reduce the asphalt smoke emissions in the heating process of asphalt to achieve the true meaning of smoke suppression and emission reduction [38]. This study is in line with the concept of harmonious coexistence of man and nature, which is valuable for improving the environmental protection and safety of asphalt pavement construction and has a far-reaching impact on ensuring the health of construction workers and promoting the development of asphalt pavement construction in the direction of non-pollution [39].

## 2. Experimental Materials and Methods

### 2.1. Raw Materials

The asphalt employed in this study is SBS asphalt, and the pertinent performance indicators are presented in Table 1. First, SBS-modified asphalt is widely used in road construction because of its good high and low-temperature performance. Therefore, it is more important to study the VOC emission of SBS-modified asphalt for practical engineering applications. Secondly, SBS itself can form a mesh structure with asphalt to immobilize small molecules, and chemical reactions occur to prevent the production of certain intermediate products, thus cutting off the VOC formation process. Then, SBS-modified asphalt releases less VOCs than matrix asphalt. Finally, the use of SBS-modified asphalt can improve asphalt road performance and, at the same time, inhibit the release of asphalt fumes. This dual effect makes SBS-modified asphalt more favorable in practical applications. The zeolite used in this study is a general term for a family of architectural water-containing silica-aluminate minerals, including naturally occurring natural zeolites and synthetic crystals, and natural zeolite has a unique skeletal structure. The technical parameters of zeolites and LDHs are shown in Table 2 and Table 3.

### 2.2. Experimental Methods

#### 2.2.1. Preparation of Modified Zeolites

The method was as follows: First, weigh 100 g of zeolite in a beaker, and 200 mL of hydrochloric acid was added to the beaker and stirred well with a glass rod, then transferred to a petri dish and stored in a sealed container for 48 h. After 48 h, it was removed and transferred to a centrifuge, where the sample was washed with deionized purified water. After washing, the wet samples were transferred to a filtration unit and filtered several times. Finally, the samples were transferred to an oven and dried at 80 °C. The samples were then dried in an oven.

#### 2.2.2. Preparation of Modified Bitumen

The mixing process of SBS asphalt and modifier used in this experiment is as follows [40]: (1) A stainless steel container is used to contain a certain volume mass of SBS asphalt, and the asphalt is heated to 160 °C in a constant temperature oven, such that the asphalt in the container is in the flow state. (2) Weigh the inhibitor based on the quality of asphalt and the pre-designed blending ratio, add the inhibitor into the hot-melted asphalt 3~4 times while stirring, and stir at a low speed of 800 r/min for 15 min at 160 °C. (3) Move the container to the high-speed emulsifying shear after the low-speed stirring, use the rotational speed of 6000 r/min high-speed shear for 40 min, and keep the temperature constant throughout the process at 160 °C, and finally obtain the homogeneous modified asphalt. (4) The SBS matrix asphalt and SBS asphalt mixed with LDHs and HCl-treated zeolite were named SBS (SBS-modified asphalt), LMA (6 wt% LDHs-modified asphalt), OMA (6 wt% hydrochloric acid pretreated zeolite-modified asphalt), and CMA (3 wt% hydrochloric acid pretreated zeolite and 3 wt% LDHs composite-modified asphalt), respectively.

### 2.3. Experimental Methodology

#### 2.3.1. Characterization Tests of Modified Zeolites

Natural and synthetic zeolites are water-containing shelf silica-aluminates. The natural zeolite pores have a strong adsorption capacity, but the natural zeolite pore channels contain impurities such as gel and sodium amphibole, which results in some channels and pores being non-connected. This affects the formation of crystalline and adsorbent water within the zeolite and reduces its specific surface area, which in turn affects the effect of its adsorption. In this experiment, the hydrochloric acid-modified zeolite was subjected to a series of analyses, including X-ray fluorescence spectroscopy (ESCALAB 250Xi, Thermo Scientific, East Grinstead, UK), field emission scanning electron microscopy (JSM-IT800, JEOL Ltd., Tokyo, Japan), and fully automated specific surface area and porosity analysis (ASAP 2020, Micromeritics Instrument Corporation, Norcross, GA, USA), in order to meet the specified test standard and to ascertain its physical and chemical properties.

#### 2.3.2. Asphalt Performance Tests

The properties of the prepared asphalt can be evaluated through the implementation of pertinent tests. The first step involved testing the three primary indicators of asphalt and viscosity to facilitate a comparative analysis of the fundamental properties of modified asphalt and SBS asphalt. This included assessing the softening point, penetration, viscosity, and ductility of both materials. Softening points are used in the global method, and the test parameters are as follows: 5 °C ± 0.5 °C constant temperature water tank, the rate of temperature increase of 5 °C ± 0.5 °C/min. The experimental parameters of the ductility are as follows: test temperature of 15 °C, stretching speed of 5 ± 0.25 cm/min, the use of glycerin talc isolate, and the proportion of 2:1. Needle penetration experimental parameters are as follows: an experimental temperature of 25 °C ± 0.1 °C, and the total mass of the standard needle, connecting rod, and additional weights were 100 ± 0.05 g. The parameters of the asphalt viscosity test are 135 °C ± 1 °C (this test temperature is based on the test temperature of SBS asphalt), rotational speed 20 r/min, and torque control in the range of 20~80%. TFOF aging is in accordance with the asphalt specification T0602 asphalt specimen preparation methods. An asphalt binder of 50 g ± 0.5 g per group was added to the sample dish, followed by the formation of asphalt thickness as a uniform film. The asphalt sample dish was placed in the asphalt rotating film oven at 135 °C (SYD-3061, Shanghai Lei Yun Test Instrument Manufacturing Co., Ltd., Shanghai, China) in the thermal oxidation of aging for 4 h. The aging process turns over once an hour until the completion of the thermal oxidation of aging. PAV aging is the TFOF aging asphalt sample dish into the pressure vessel, in the pressure of 2.1 MPa ± 0.1 MPa, temperature of 50 °C under the condition of aging for 20 h until the aging is completed. Temperature scanning test trials were then carried out on the modified asphalt as well as the aged, modified asphalt, using a dynamic shear rheometer to measure factors including complex modulus, phase angle, rutting factor, and fatigue factor (Anton Paar, MCR101, Anton Paar GmbH, Graz, Austria). The parameters of the DSR test were as follows: rotor diameter of 25 mm, plate gap of 1 mm, temperature increase rate of 2 °C/min, frequency of 10 rad/s. Furthermore, in consideration of the low-temperature performance of the modified asphalt, the bending creep stiffness and creep rate of the various asphalts were evaluated using the BBR (XYD-0627, Sigma-Aldrich, St. Louis, MO, USA), thereby reflecting the rheological properties of the different asphalts. Subsequently, thermogravimetric analysis was conducted on the modifiers and modified bitumen, with the objective of measuring and analyzing the high-temperature stability of modified zeolites and LDHs. Finally, the infrared effect of the modified asphalt was measured using the Fourier Transform Infrared (FTIR) test (Nicolet 6700, Thermo Fisher Scientific, Waltham, MA, USA), with the aim of analyzing the differences between the modified asphalt and the matrix asphalt through infrared spectroscopy.

#### 2.3.3. Release Behavior of Volatile Organic Compounds (VOCs)

The composition of asphalt fumes generated by heating is also more complex due to the numerous substances present in asphalt. It is challenging to construct a device that accurately simulates the collection of asphalt fumes in a laboratory setting, given the complexities involved in the construction environment. The proposed device comprises four main steps: heating the asphalt, stirring the asphalt, filtering the fumes, and collecting the fumes. After an extensive literature review, a gas collection device was finally developed. The parameters of the GC-MS test were as follows: the experimental temperature was 163 °C (temperature increase phase), the holding time was half an hour (VOCs generation phase), and the rate of the gas sampling pump was 500 mL/min (collection phase), and the thermal desorption temperature was 120 °C (VOCs analysis phase). Firstly, a temperature sensor and stirrer are connected to a three-necked flask filled with asphalt. Subsequently, a conical flask filled with a cyclohexane solution is connected to a conical flask with a filter, which is then extracted by a gas sampling pump through a conduit to a PTFE sampling bag. The VOCs gas is then sucked on an adsorption conduit, which can be used for gas chromatography and mass spectrometry (GC-MS) testing. This method is employed for the qualitative analysis of VOCs by means of the GC-MS instrument. A comparison of the results with the data in the mass spectrometry library allows the peaks produced by the corresponding substances in the chromatogram to be identified visually. Subsequently, the VOCs collected in the tetrafluoroethylene sampling bag are separated from the requisite components by gas chromatography, diluted, and then subjected to quantitative detection of the substances.

## 3. Results and Discussion

### 3.1. Composition Comparison Before and After HCl Pretreatment of Zeolite

**Figure 1:**
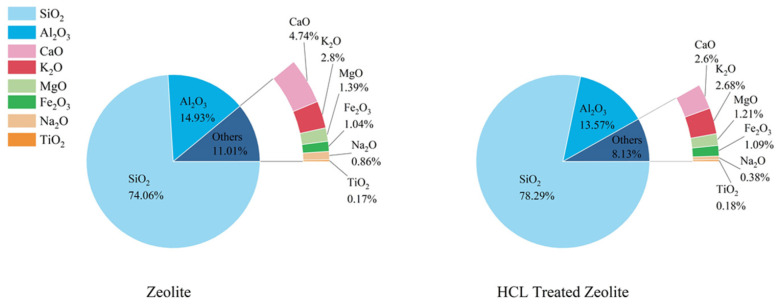
Compositional analysis of zeolite pretreatment.

According to the analysis of XRF experimental data, its composition is shown in Figure 1. Natural zeolite is a water-containing shelf silica-aluminate, but the pore channels of natural zeolite contain impurities such as gel and sodium amphibole, which makes part of the channels and the pores are not connected, which affects the formation of water of crystallization and adsorbed water inside the zeolite, resulting in the natural zeolite has a low water content (less than 10%). Treatment with hydrochloric acid solution can change the composition of impurities in zeolite. Cl^−^ in hydrochloric acid reacts with cations in zeolite, mainly changing the composition of Al^3+^, Ca^2+,^ and Na^+^. The main structure of zeolite is a silica-aluminate structure; the reaction with hydrochloric acid will change its structure, and the Al^3+^ precipitation phenomenon occurs. The precipitation of Ca^2+^ is a consequence of the interaction between zeolite and hydrochloric acid, which is attributable to the carbonate structure of the former. This reaction results in the formation of a soluble state of Ca^2+^, which can be removed through the rinsing of the treated zeolite. Na^+^ exists in the pore diameter of the zeolite as sodium amphibole and produces precipitation by the reaction with hydrochloric acid. Si and Al are the main constituents in zeolite, and the relative content of Si increases as Al decreases before and after hydrochloric acid treatment. The removal of these impurity elements results in an increase in the Si/Al ratio in zeolites.

Macroscopically, the zeolite, following treatment with hydrochloric acid, exhibited alterations in the silica-aluminum ratio and skeleton structure. However, the impurities within the pores were removed, and the internal pores were enlarged, resulting in an increased specific surface area and water absorption rate. Additionally, the adsorption performance was enhanced.

### 3.2. FT-IR Analysis of Different Modifiers and Modified Bitumen

**Figure 2:**
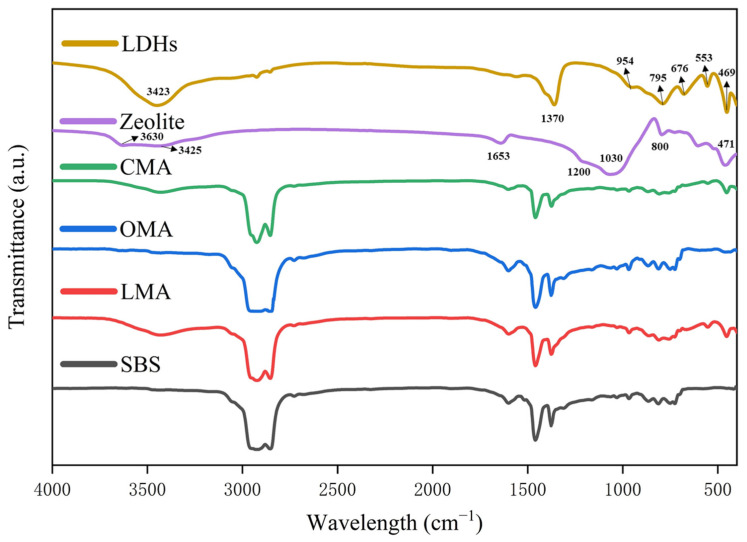
Infrared spectra of modified bitumen.

Figure 2 illustrates the infrared spectra of modified zeolites, LDHs, and bitumen. In the IR curve of LDHs, LDHs showed an interlayer ${CO}_{3}^{2-}$ absorption peak near 1370 cm^−1^ in the infrared curve of LDHs, there are strong characteristic absorption peaks near 1370 cm^−1^, and there are characteristic absorption peaks of M-O bond and interlayer anion on the laminates in the range of 1000~400 cm^−1^; its infrared absorption range can be changed by modifying the composition. A comparison of the peak appearing near 795 cm^−1^ with the IR profile of zeolite indicates that LDHs are more structurally ordered than zeolites.

In the infrared spectrum of zeolite, the absorption peak near 1030 cm^−1^ is clearly discernible and can be attributed to the characteristic peaks of Si-O-Si symmetric and asymmetric stretching vibrations. Similarly, the absorption peak in the vicinity of 1200 cm^−1^ is ascribed to the Si-O antisymmetric stretching vibration. The distinctive peak observed near 800 cm^−1^ is associated with Al-O. Additionally, the characteristic peaks appearing at 471 cm^−1^ are attributed to Si-O as well as O-Si-O antisymmetric bending vibrations. The strong and broad absorption peak at 3425 cm^−1^ corresponds to the characteristic peak of -OH stretching vibration, indicating the condensation of physically adsorbed water within the pores of zeolite. Similarly, the absorption peak at 3630 cm^−1^ corresponds to the presence of bound water in the zeolite.

However, in the infrared spectra of SBS asphalt and modified asphalt, the main functional groups corresponding to the characteristic peaks are methyl-CH_3_, methylene-CH_2_, benzene ring, sulfinyl S=0, and carbon-carbon double bond C=C. The characteristic peaks near the wavelengths 2919 cm^−1^ and 2850 cm^−1^ have strong absorption, sharp waveforms, and narrow peak widths, which are the result of the expansion and vibration of methylidene CH. The frequency of the asymmetric angular vibration of CH is 1460 ± 5 cm^−1^, and the symmetric angular vibration frequency is 1375 ± 5 cm^−1^ in the figure. Methyl CH_2_ expansion vibration results. CH_3_ asymmetric angular vibration frequency of 1460 ± 5 cm^−1^, symmetric angular vibration frequency of 1375 ± 5 cm^−1^, 1455 cm^−1^ and 1376 cm^−1^ position of the two characteristic peaks, that is, CH_3_ angular vibration caused by; in addition, the wave number of 1600 cm^−1^ also has a more pronounced peaks, which is the benzene ring skeleton of the expansion and contraction of the vibration caused by. Compared with SBS asphalt, the infrared curve of modified asphalt did not show new absorption peaks. Therefore, no chemical reaction was generated by the mixing of LDHs and zeolite modifiers with SBS asphalt.

### 3.3. Effect of Different Modifiers on the Basic Properties of Asphalt

**Figure 3:**
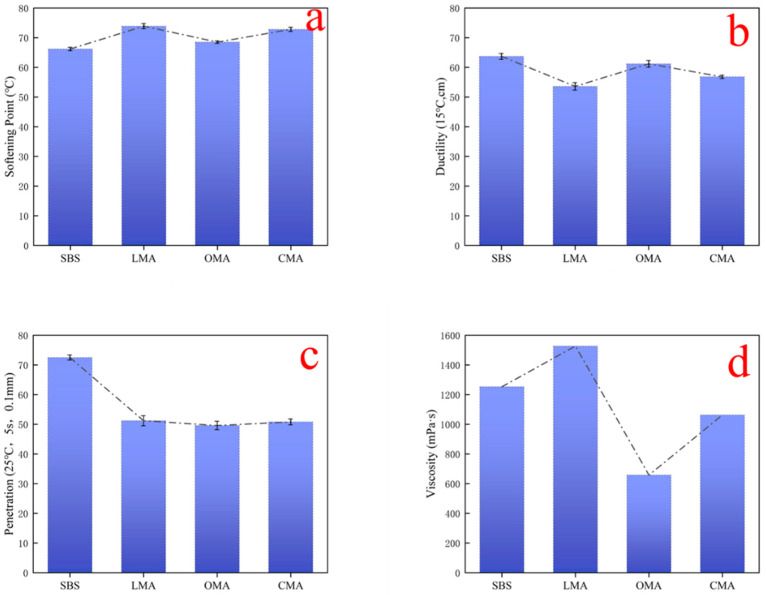
The basic properties of the four types of asphalt are (**a**) softening point, (**b**) ductility, and (**c**) permeability. (**d**) viscosity.

Figure 3 shows the physical properties of asphalt after modification. Compared to SBS asphalt, the softening point and viscosity of LMA are elevated, but the needle penetration and ductility are decreased, whereas the ductility and viscosity of CMA are decreased. This indicates that the addition of LDHs and HCl-treated zeolite changes the high and low-temperature properties of the modified asphalt. However, LMA possesses higher viscosity due to the fact that the remaining macromolecules require a certain amount of energy to move in the asphalt. The compounded CMA asphalt samples also have the same two characteristics mentioned above. For SBS asphalt, which has a high viscosity, the addition of LDHs leads to an increase in the viscosity of the asphalt, which is due to the inhibition of the movement of the asphalt binder by the LDHs, but the addition of HCl-treated zeolite significantly reduces the viscosity of the asphalt. For the asphalt sample OMA, the addition of HCl-treated zeolite will greatly improve the problem of elevated viscosity of asphalt binder and poor workability in construction caused by the addition of LDHs to SBS asphalt, and improve the fluidity of asphalt, as well as reduce the energy consumption in practical applications. According to the Superpave specification, all the samples met the specification, indicating that they can still be fully utilized during construction.

### 3.4. Effect of Different Modifiers on the High-Temperature Rheological Properties of Asphalt

#### 3.4.1. Complex Modulus (G*) and Phase Angle of Different Asphaltenes

**Figure 4:**
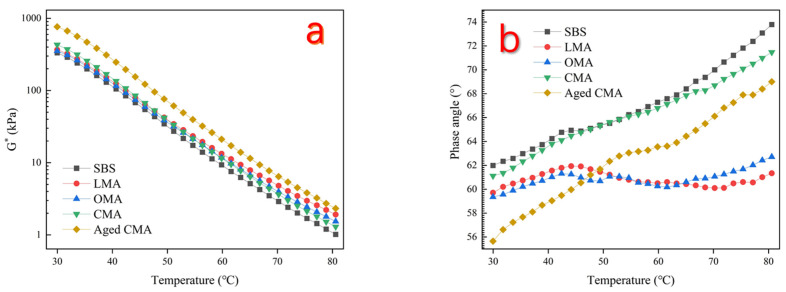
(**a**) Complex modulus of the five asphalts; (**b**) Phase angle of the five asphalts.

Figure 4 shows the G* and δ data of the four asphalts at 30–80 °C. As the temperature increases, the complex modulus (G*) on all four asphalts decreases, indicating that the asphalt binder softens with increasing temperature. This is the reason why asphalt roads will develop rutting marks under high-temperature exposure in summer. The maximum complex modulus of CMA asphalt was 430.34 kPa at a temperature of 30 °C, indicating that the optimal enhancement in the flow properties of SBS asphalt was achieved through the combination of hydrochloric acid-modified zeolites and LDHs at moderate temperatures. The complex modulus of both LMA and OMA is higher than that of SBS asphalt, indicating that both modifiers contribute to the hardening of the latter. The data also show that when the proportion of the two modifiers is fixed, the increase in the complex modulus of asphalt caused by LDHs is more significant. On the other hand, at temperatures of 30–80 °C, the phase angle of the four types of asphalt increases with increasing temperature. In particular, after the incorporation of zeolite and LDHs, the phase angle of the modified asphalt is lower than that of SBS asphalt, and it fluctuates in a wave-like pattern. Conversely, the complex modulus of aged CMA asphalt at 60 °C exhibited a notable increase from 11.508 kPa to 21.145 kPa, accompanied by a reduction in phase angle from 66.79° to 63.55°. These observations suggest that the rheological properties of the aged CMA bitumen are likely to deteriorate as a consequence of the enhanced bitumen hardness resulting from the aging process. The data from CMA asphalt indicate that the composite blending of hydrochloric acid-modified zeolites and LDHs can enhance the high-temperature rutting resistance of SBS asphalt and mitigate the deterioration of the high-temperature rheological properties of modified asphalt.

#### 3.4.2. Rutting Factor for Different Asphalt

**Figure 5:**
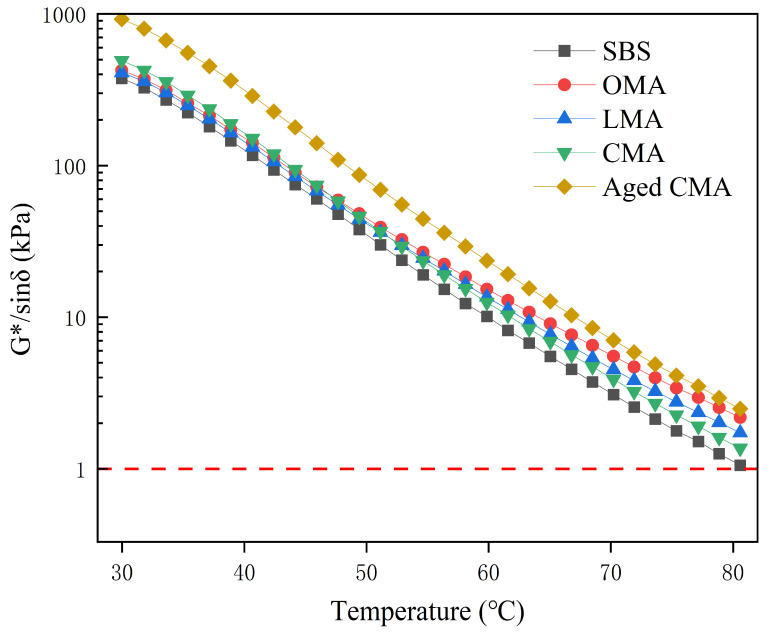
High-temperature rutting factor (Rf) for five asphalts.

The resistance of asphalt to permanent deformation is expressed by the rutting factor (Rf). Figure 5 shows the rutting factors of four types of asphalt. In general, the higher the rutting factor of asphalt, the stronger its rutting resistance. In Figure 5, the rutting factors of the four types of asphalt all decrease significantly with increasing temperature, indicating that the asphalt may have changed from a viscous state to an elastic state. In the temperature range of 30–80 °C, the rutting coefficient of 10.119 kPa for SBS asphalt at 60 °C is lower than that of the other three modified asphalts. This is evidenced by the differing Rf values between the asphalts. Furthermore, the differences are becoming increasingly pronounced, which suggests that the resistance to permanent deformation of the various modifiers is influenced by the permanent deformation. The LDHs, hydrochloric acid-modified zeolite, and its two modifiers are combined to enhance the rutting resistance performance. From the rutting coefficients of CMA asphalt before and after TFOT aging, it can be observed that the Rf value of 924 kPa at 30 °C is 432.72 kPa higher than that of the unaged CMA asphalt. Furthermore, the R-value of the rutting coefficient of the aged asphalt at 80 °C is 1.12 kPa higher than that of the unaged CMA asphalt, which demonstrates that the aged asphalt exhibits enhanced rutting resistance within the temperature range of 30–80 °C and that its properties remain stable. According to the SHRP proposed asphalt pavement performance specification standard, the rutting factor of asphalt, that is, the G*/sinδ value, must be greater than 1 kPa, and the average maximum pavement design temperature at this time is the high-temperature grade. The rutting coefficient at 81 °C indicates that the Rf of SBS asphalt is greater than 1.0 kPa, thereby meeting the standard. Furthermore, the Rf of other asphalt is higher than that of SBS asphalt, which substantiates the assertion that the incorporation of hydrochloric acid-modified zeolite and the two modifiers of LDHs can markedly enhance the rutting resistance of SBS asphalt.

#### 3.4.3. Fatigue Factor for Different Asphalts

**Figure 6:**
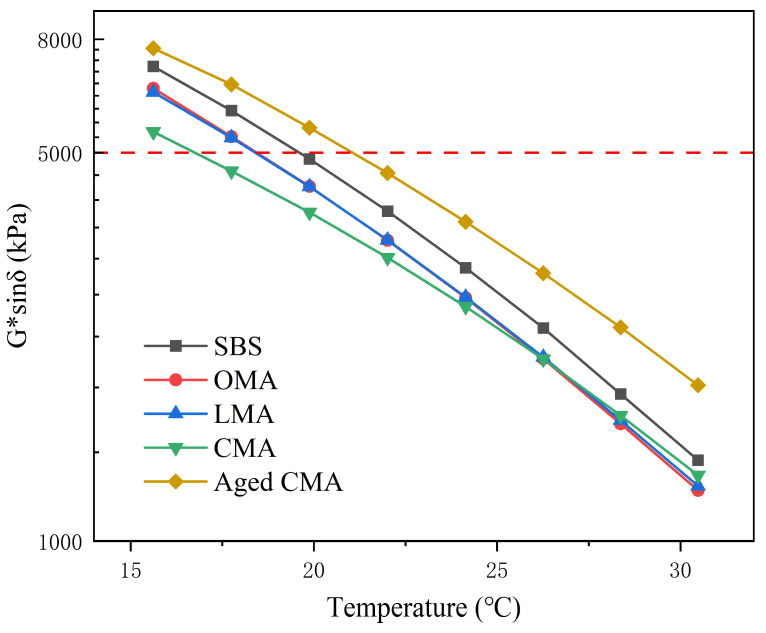
Fatigue factor for five types of asphalt.

According to the SHRP standard for asphalt pavement performance specifications, in order to meet the fatigue cracking resistance of asphalt, the fatigue factor (Ff) of asphalt, i.e., G*sinδ, must be less than 5000 kPa. As shown in Figure 6, the Ff of the four types of asphalt decreased significantly with increasing temperature in the temperature range of 15–30 °C. Moreover, at moderate temperatures, the fatigue factor tends to approach the standard, indicating that the temperature at which asphalt is prone to fatigue cracking tends to approach this temperature. It is worth noting that due to the action of hydrochloric acid-modified zeolite, LDHs, and their blends, the Ff of the modified asphalt is lower than that of the SBS asphalt. Figure 6 illustrates the fatigue factor of CMA asphalt in the temperature range of 15 °C to 30 °C, both prior to and following TFOT aging, as well as PAV aging. The Ff value of CMA is 7707.2 kPa, which is 2255.4 kPa higher than that of CMA asphalt after aging at 15 °C and 1907.1 kPa, which is 595.2 kPa higher than that of CMA asphalt after aging at 30 °C. This is attributable to the hardening of the binder following TFOT and PAV aging, which results in a notable reduction in the fatigue resistance of the asphalt post-aging. The above shows that hydrochloric modified zeolites and LDHs improve the fatigue resistance of asphalt at moderate temperatures, and that of all modified asphalts at 15–30 °C, only SBS asphalts that are compounded with hydrochloric modified zeolites and LDHs can meet the 17 °C criterion.

### 3.5. Effect of Different Modifiers on the Low-Temperature Rheological Properties of Asphalt

**Figure 7:**
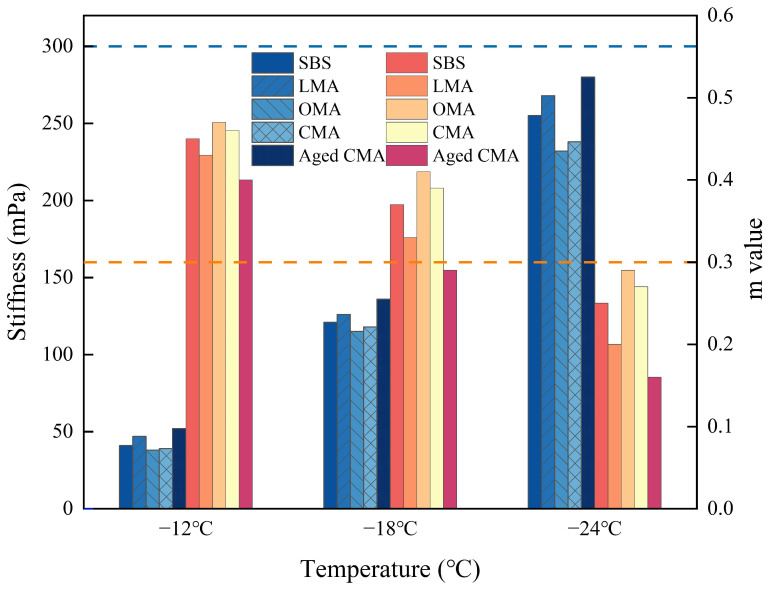
Low-temperature rheological properties of five asphalts.

The S and M values of the four asphalts at different temperatures are shown in Figure 7. The addition of modifiers within the specified range of asphalt increases the S value of asphalt as a whole. It can be known that S keeps increasing as the temperature decreases. The greater the S, the lesser the creep flexibility, which indicates an increase in the hardness of the asphalt at that temperature. When the asphalt specimens are at low temperatures, the flow characteristics are restricted, and cracking is easily caused. The S-values of SBS asphalt were observed to be 4.91%, 10.56%, and 8.65% higher than those of CMA at −12 °C, −18 °C and −24 °C, respectively. This indicated that the addition of the modifier resulted in an improvement in the fluidity properties of SBS asphalt, particularly in the rheological properties of OMA asphalt, which were found to be more excellent at −24 °C to −12 °C. The M value characterizes the time sensitivity of the stiffness of the asphalt and its ability to relax internal stress. Figure 7 illustrates that the M-value of OMA is the highest and that of LMA is the lowest at −18 °C. This suggests that the incorporation of hydrochloric acid-modified zeolites into SBS asphalt is the most effective method for enhancing its low-temperature cracking resistance. Conversely, the addition of LDHs alone has a detrimental impact on the low-temperature cracking resistance of SBS asphalt. It is regrettable that the low-temperature rheological properties of the aged CMA asphalt were poor, such that it failed to reach the M-value criterion at both −18 °C and −24 °C. The greater the m value, the better the stress relaxation ability of the asphalt and the stronger its resistance to low-temperature cracking. In the experiments conducted at temperatures between −12 °C and −24 °C, the S-value of SBS increased from 41 mPa to 255 mPa, while the M-value decreased by 45.22%. Conversely, the S-value of CMA increased from 39 mPa to 232 mPa, and the M-value decreased by 41.3%. Furthermore, the incorporation of hydrochloric acid-modified zeolites in SBS asphalt results in enhanced low-temperature cracking resistance. However, the addition of LDHs has the opposite effect, leading to a weakening of the low-temperature rheological properties of SBS asphalt. The aforementioned findings indicate that hydrochloric acid-modified zeolites and LDHs exert minimal influence on the low-temperature resistance of bitumen at moderate temperatures. Moreover, none of the four bitumen samples demonstrated compliance with the standard requirements at −24 °C.

### 3.6. Effect of Different Modifiers on the Emission Characteristics of VOCs

#### 3.6.1. Analysis of the VOC Composition of Different Asphalt

This study used the GC-MS method to determine the smoke generated during the heating of asphalt. The effect of LDHs, hydrochloric acid-modified zeolites, and their complex blending on the release of VOCs from asphalt binders was analyzed from the detection of nearly 100 VOC components. This study divided VOCs into alkanes, alkenes, aromatics, and other organics.

**Figure 8:**
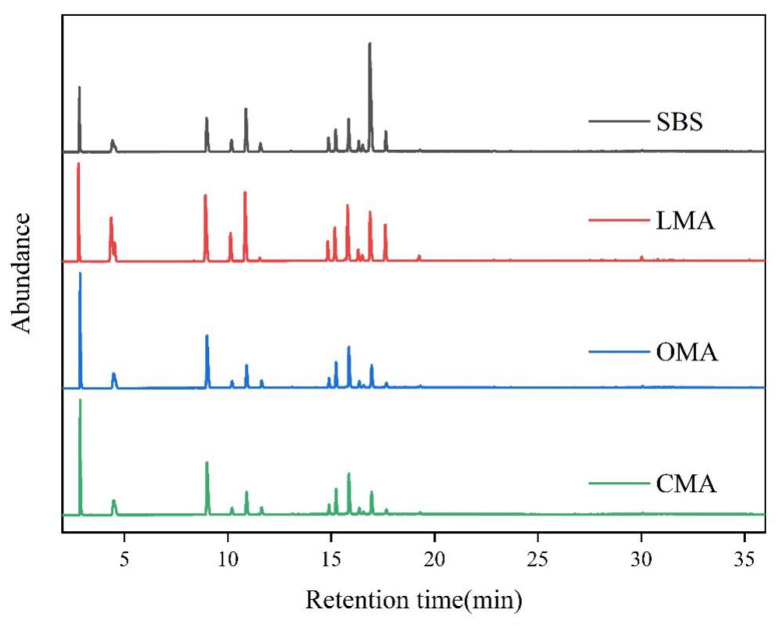
VOCs composition of four types of asphalt settling time.

Figure 8 shows the GC-MS chromatograms of SBS asphalt and the addition of LDHs, zeolites, and their complex doping inhibitors. The location of the peaks in the figure represents the production of organic matter in the siphoning process, and the amount of VOC substances produced can be clearly expressed visually from the increased intensity of the peaks in the spectrum. The size of the intensity peaks in the graph represents the content of the substance; the stronger the peak intensity, the more ions are detected and the stronger the signal. The position of the main peak is clearly observed in the first 18 min from the graph. From Figure 8, it can be seen that with the addition of zeolite inhibitors, the number of peaks decreases significantly, and the intensity of peaks is relatively weakened; however, with the addition of LDH inhibitors, the number of peaks and the intensity of peaks do not change too much relative to SBS asphalt. With the addition of LDHs and zeolite, the number of peaks decreased by 7.24% and 52.67%. It can be calculated that when the inhibitor was changed from LDHs to zeolite, the number of peaks decreased by 45.43%. The main reason for this is that zeolite has a better adsorption pore size to absorb VOCs, while LDHs just play a role in isolation and do not adsorb and immobilize VOCs. Meanwhile, it can be found that the number of peaks of CMA in the figure is significantly reduced, and the intensity is obviously weakened, compared with the number of peaks of SBS, which is reduced by nearly 61.6%, which indicates that the LDHs and hydrochloric acid modified zeolite compound blending added into SBS asphalt has the best emission reduction effect.

#### 3.6.2. VOCs Composition Analysis of Different Asphalt

**Figure 9:**
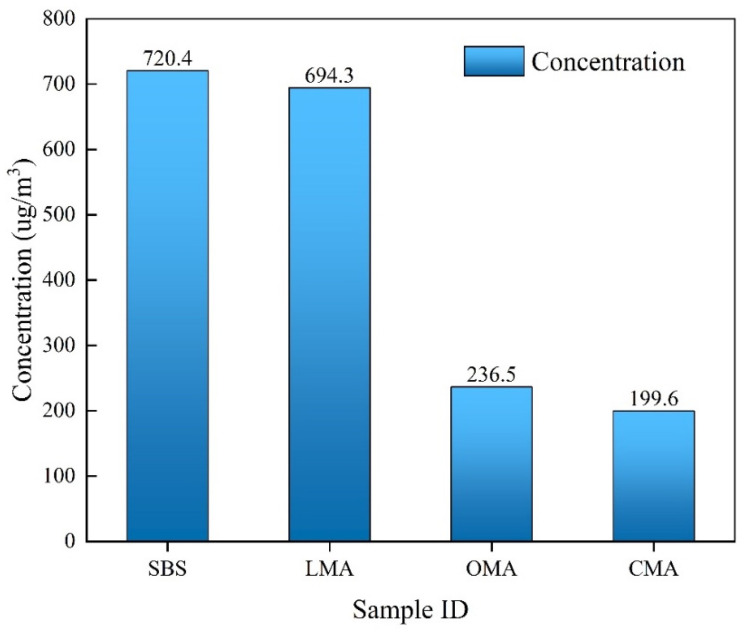
Total VOC emissions from four types of asphalt.

**Figure 10:**
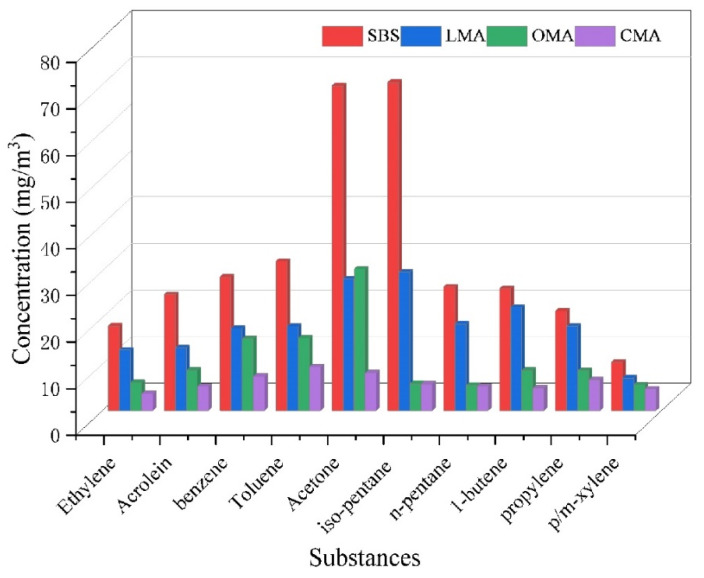
Concentrations of the 10 highest concentrations of VOCs.

Nearly 80 high-concentration VOCs were determined by GC-MS experiments to study the inhibitory effects of different modifiers on different types of VOCs. Figure 9 shows the analysis of the total VOC concentration produced by the four types of asphalt. It can be seen that the VOC production of SBS asphalt is about 2.95 times that of OMA and 3.61 times that of CMA. Table 4 shows the specific composition table of VOCs. The combination of the chromatogram and the composition table can be clearly observed in the pattern of VOC generation as well as the types. Therefore, the synergistic effect of the zeolite modifier on asphalt fume reduction can be found to be significant. As shown in Figure 10, the 10 VOCs with the highest concentrations were analyzed according to the highest VOC concentration ranking, in which alkanes and olefins occupied the main position, and the VOC concentrations of the four asphalt species were ranked as SBS > LMA > OMA > CMA. Combined with the qualitative analysis, it can be seen that adding LDHs, hydrochloric acid-modified zeolite, and complexes to asphalt binder can significantly reduce the VOCs released from asphalt, which mainly reduces the content of alkanes and olefins, which are the two main components, in order to achieve the effect of reducing the emission of VOCs.

As shown in Figure 10, the 10 substances with the highest concentration of releases among nearly 80 VOCs were further analyzed. From the figure, it can be seen that alkanes and olefins have the highest content, about 70 percent. From the figure, it can be found that the mixing of zeolites and LDHs will affect the production of the top ten substances of VOCs as a whole, and their specific compositions are basically the same, but their contents become relatively less overall, and the overall concentration of each substance is on a decreasing trend, which can be shown to have an inhibitory effect on VOCs.

### 3.7. The Mechanism of Action of Different Modifiers in Inhibiting VOCs Emissions

#### 3.7.1. Adsorption Effects

**Figure 11:**
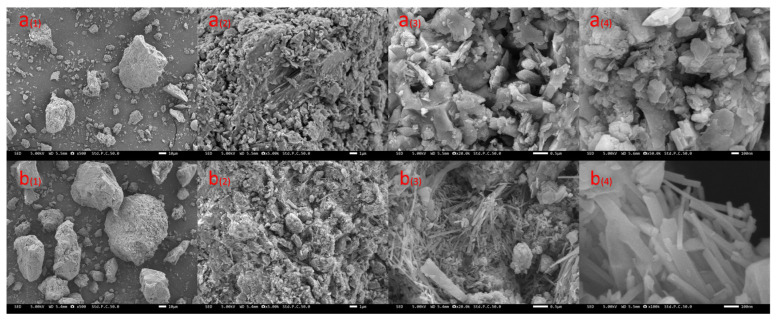
(**a_(1)_**–**a_(4)_**) Scanning electron microscope image of zeolite; (**b_(1)_**–**b_(4)_**) scanning electron microscope image of zeolite after treatment with hydrochloric acid.

**Figure 12:**
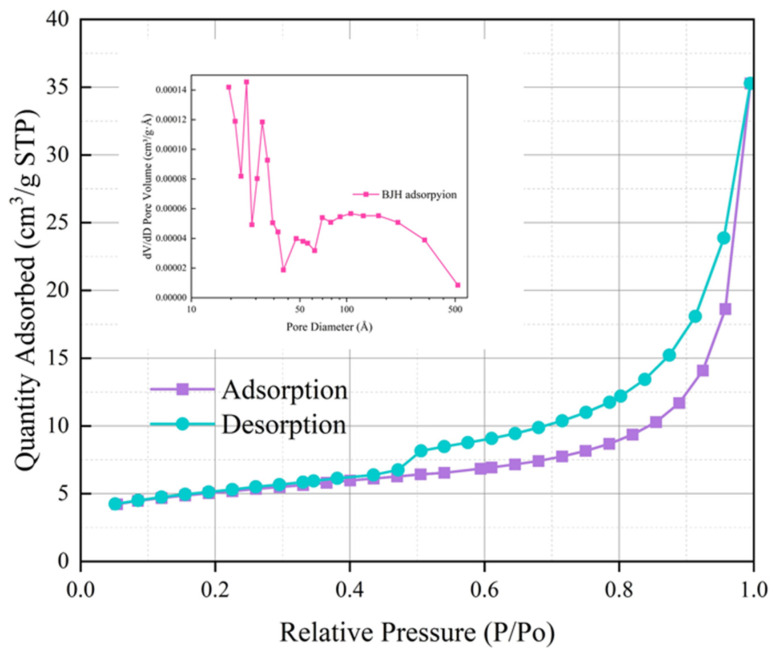
N_2_ adsorption and desorption curves of zeolite.

Figure 11a_(1)_–b_(4)_ shows the microstructures of zeolites and hydrochloric acid-modified zeolites under scanning electron microscopy. LDHs present a plate-like structure, which isolates the VOC gases and prevents them from volatilizing. On the other hand, rhombohedral zeolites present fine strips with obvious and regular porous structures attached to their surfaces, such that the adsorbed VOC gases are immobilized in the pores through intermolecular forces. Zeolites have a better porous structure for capturing VOC gases compared to LDHs. The N_2_ adsorption curve of zeolite consists of an adsorption curve and a desorption curve, as shown in Figure 12. An adsorption curve is said to be Type IV if it has a relatively high P/P_0_ at a point where a platform appears or where the last turn of the isotherm ends. The presence of mesopores (0–20 nm) in the zeolite is indicated by the appearance of a Type IV adsorption curve.

Zeolites adsorb monolayers at relatively low P/P_0_ values. As the gas is adsorbed, the adsorption does not stop when the zeolite surface is covered with an atomic thickness of gas. On the other hand, as the relative pressure P/P_0_ value increases, the gas continues to be adsorbed, eventually forming multiple layers of gas encapsulated on the surface of the zeolite, which eventually liquefies as the pressure increases. This series of behaviors illustrates the phenomenon that the porous structure of zeolite has an adsorption and immobilization effect on VOCs, and the adsorption effect is better. Therefore, the utilization of LDHs as a flame-retardant layer to immobilize VOCs, coupled with the incorporation of zeolite as a porous structure to adsorb and immobilize these same gas molecules, and the addition of the composite modifier to asphalt, has the potential to exert a considerable inhibitory effect on VOCs.

**Figure 13:**
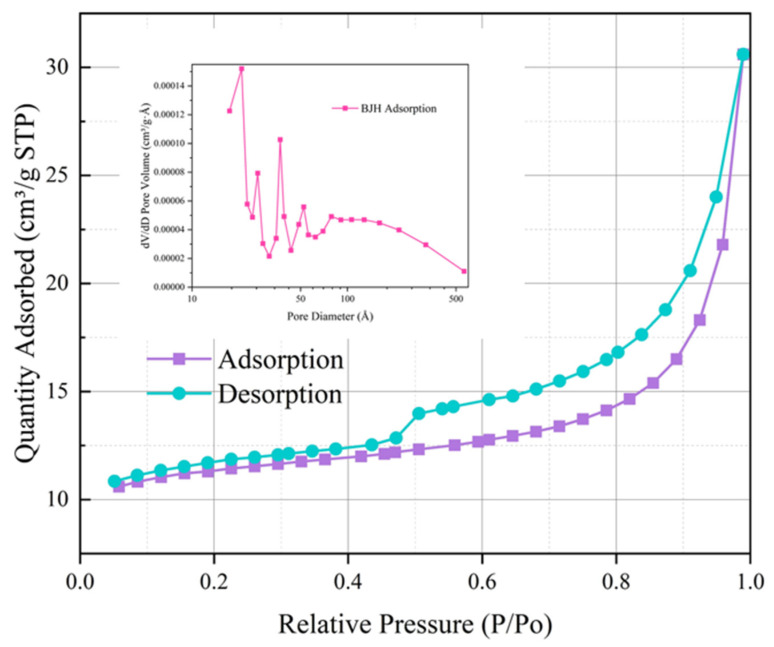
N_2_ adsorption and desorption curves of HCl-treated zeolite.

In addition, we can see, according to Figure 13, that the specific surface area of zeolite modified by hydrochloric acid increases, the pore volume becomes larger, etc., and these changes are favorable for VOC adsorption. It can also be seen that the hydrochloric acid-modified zeolite adsorption curve is also Type IV. Then, by observing the zeolite image in a scanning electron microscope, it can be found that there is strong adsorption in zeolite, which makes the crystal shape of zeolite presenting a fine strip, and it can effectively adsorb the VOCs gas to be fixed in the pore space with an uneven surface.

#### 3.7.2. Flame Retardant Effect

**Figure 14:**
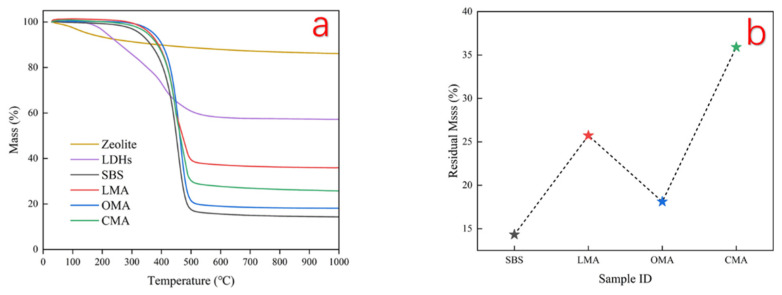
(**a**) TG curves of the four asphalts; (**b**) residual mass of the four asphalts.

**Figure 15:**
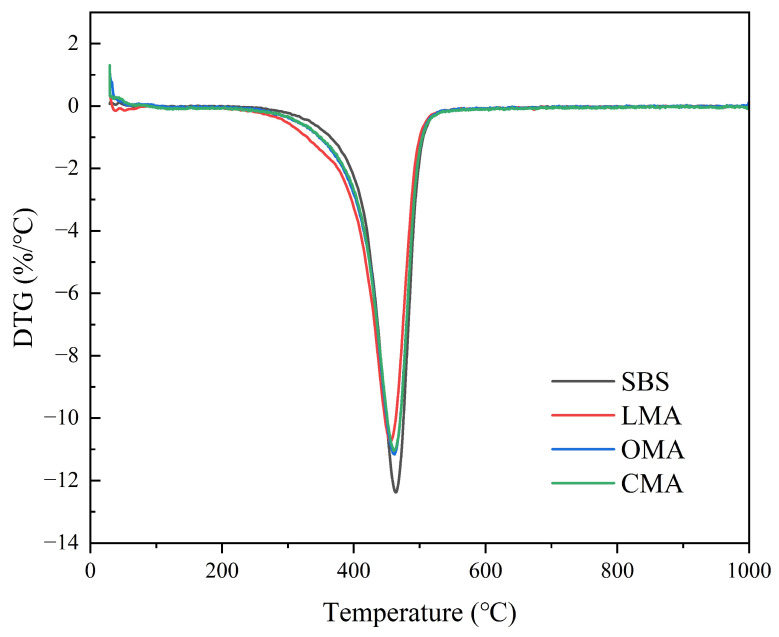
DTG curve of all binders.

According to the compositional analysis of zeolite pretreatment in Figure 1, the content of Al_2_O_3_ and MgO was reduced after hydrochloric acid treatment. Therefore, hydrochloric acid treatment negatively affected the flame retardation. Therefore, the addition of LDHs can reduce the volatilization of asphalt flammable volatiles and play an isolating role. As shown in Figure 14, the TG curves show the mass loss of asphalt during the combustion process, in which SBS asphalt has the largest mass change, followed by OMA, with less loss of LMA and CMA, from which it can be found that the flame-retardant effect of zeolite and LDHs compounding is the best. LDHs mainly occur through the combustion of asphalt surfaces to promote the generation of dense oxidized layers with a flame-retardant effect. Zeolite contains bound water, structural water, and free water that can absorb heat in the process of asphalt combustion into water vapor while reducing the temperature of asphalt combustion, thereby reducing the emission of harmful gases that play a role in diluting the effect of harmful gas concentrations. DTG curve as shown in Figure 15, with the addition of LDHs and zeolite, SBS asphalt’s maximum DTG value of the temperature from 463.7 °C to 455.5 °C, the maximum DTG value of the temperature from 461.7 °C, especially when LDHs and zeolite were compounded into the asphalt, the maximum DTG value temperature became 460.4 °C. Furthermore, it can be seen from the thermogravimetric curves that the mass change of SBS is the most obvious, the mass change of CMA is the smallest, and the addition of an inhibitor can significantly reduce the mass change of asphalt. Meanwhile, LDHs and hydrochloric acid-modified zeolites can absorb heat and block smoke during asphalt heating to prevent the release of VOCs. In addition, the possible reason for the weight gain at the initial stage of the thermogravimetric curve is due to the decrease in the density of the gases around the crucible when heated and the decrease in buoyancy resulting in weight gain.

## 4. Conclusions

This study is based on the exploration of the basic properties of LDHs and hydrochloric acid-treated zeolite materials, which were applied to the treatment of SBS asphalt flue gas due to their excellent performance in gas adsorption. The effects of LDHs and hydrochloric acid-treated zeolites on the basic properties, high and low-temperature properties, and VOCs adsorption properties of SBS asphalt were studied. The following conclusions were drawn from the above analysis:

- HCL-treated zeolite, an adsorbent material with a high specific surface area, high porosity, and structural order, was prepared through the study of plagioclase zeolite, which increased its specific surface area from 17.9066 m^2^/g before acid modification to 41.2528 m^2^/g.
- The incorporation of layered double hydroxides (LDHs) and hydrochloric acid-treated zeolites into styrene-butadiene-styrene (SBS)-modified bitumen significantly alters its basic properties. This modification increases the softening point, decreases the needle penetration and ductility, and slightly reduces the fatigue resistance. In terms of resistance to permanent deformation, the introduction of LDHs, hydrochloric acid-modified zeolites, and their complexes significantly improved the rutting resistance of SBS asphalt in the temperature range of 30 °C to 80 °C. It is noteworthy that the Rf values of these modified asphalts were consistently higher than those of unmodified SBS asphalts. On the contrary, the Ff values of the SBS asphalts were consistently higher than those of the other three asphalts in the same temperature range, suggesting that the incorporation of LDHs and hydrochloric acid-modified zeolites enhanced the fatigue resistance of the asphalts. In particular, the Rf and Ff data indicate that the aged CMA has significantly higher values than the other four types, a result attributed to the combined effect of TFOT aging and binder hardening after PAV aging, which leads to a reduction in rheological properties and a significant decrease in the fatigue resistance of the aged bitumen. The S and M values of both SBS and CMA asphalts changed in the low-temperature range of −12 °C to −24 °C. The S value of SBS asphalt increased from 41 mPa to 255 mPa with a decrease in M value of 45.22%, while the S value of CMA increased from 39 mPa to 232 mPa with a decrease in M value of 41.3%. This indicates that there is less change in the properties of CMA. Overall, the addition of hydrochloric acid-modified zeolites and LDHs to SBS asphalt not only enhanced its rutting and fatigue resistance but also improved its rheological properties at high temperatures. Meanwhile, the addition of hydrochloric acid-modified zeolites alone to SBS asphalt improves its low-temperature cracking resistance, while the addition of LDHs decreases the low-temperature rheological properties of SBS asphalt.
- Gas chromatography-mass spectrometry (GC-MS) analysis of the samples showed that the total VOCs emitted from LMA, OMA, and CMA asphalt were significantly reduced by 3.6%, 67.2%, and 72.2%, respectively, when compared to virgin SBS asphalt. The results indicated that the VOC molecules in CMA asphalt were effectively adsorbed by LDHs and the porous structure of hydrochloric acid-modified zeolite material, which resulted in a significant reduction in asphalt fume emissions. It is noteworthy that they were both effective in reducing VOC emissions, and we found that the adsorption capacity of hydrochloric acid-modified zeolite was significantly better than that of LDHs.
- The inhibition of asphalt fumes by hydrochloric acid-modified zeolites and LDHs is influenced by a number of factors, including temperature, environmental conditions, and specific types of inhibitors. Microscopic examinations have shown that hydrochloric acid-treated zeolites and LDHs can reduce the risk posed by asphalt fumes by reducing the release of VOCs through adsorption and confinement mechanisms. Reducing the emission of VOCs is a key step in improving the safety and environmental sustainability of asphalt applications.
- In future research, the road performance of this material applied to asphalt should be further investigated to verify the release of VOCs in the context of the actual road construction environment.

The objective of this study is to reduce air pollution and protect human health. It is a long way to promote asphalt pavement construction towards non-pollution.

## Figures and Tables

**Table 1:** Technical parameters of SBS-modified asphalt binder.

Test Items	Units	Test Results	Technical Indicators	Test Methods
Penetration index (25 °C, 100 g, 5 s)	0.1 mm	75.5	60–80	T0604-2011
Soften point	°C	52.6	≥30	T0606-2011
Ductility (15 °C, 5 cm/min)	cm	68.6	≥65	T0605-2011
Viscosity (165 °C)	Pa-s	1.498	≤3	T06025-2011

**Table 2:** Technical parameters of zeolite.

Information	Units	Test Results
Density	kg/m^3^	2034.1
Crushing rate	%	0
Wear rate	%	0
Bulk density (tapped)	kg/m^3^	954.4
Natural bulk density	kg/m^3^	827.2
Void fraction	%	59.3
Apparent density	kg/m^3^	2034.1
Ammonia absorption value	mmol/L	168.84
Loss on drying	%	3.61
Hydrochloric acid solubility	%	3.04
MgO/Al_2_O_3_	-	4.0~5.0
Heat loss	%	≤1.5
Bulk density	g/cm^2^	0.2~0.4
Heavy metals (lead)	ppm	≤10.0

**Table 3:** Technical parameters of LDHs.

Information	Units	Results
MgO/Al_2_O_3_	-	4.0–5.0
Heating loss	%	≤1.5
Bulk density	g/cm^3^	0.2–0.4
Heavy metal (lead)	ppm	≤10.0

**Table 4:** Technical parameters of SBS-modified asphalt binder.

Materials Style	VOCs Style	SBS (μg/m)^3^	LMA (μg/m)^3^	OMA (μg/m)^3^	CMA (μg/m)^3^
Alkane	ethane	7.35	35.66	4.83	3.62
	propane	21.34	50.40	8.59	10.56
	Isobutane	6.93	16.06	3.33	1.69
	n-butane	25.48	72.94	8.23	9.46
	cyclopentane	3.47	6.46	0.81	1.59
	isopentane	70.47	29.73	5.77	5.67
	pentane	26.55	18.58	5.40	5.20
	2,2-dimethyl butane	0.71	0.99	0.12	0.21
	3-methyl pentane	17.64	13.07	3.20	3.32
	n-hexane	18.04	36.22	5.18	10.16
	2,4-dimethylpentane	3.65	1.61	0.93	0.55
	methyl cyclopentane	14.93	10.87	3.31	3.39
	2-methyl hexane	10.57	9.79	3.31	3.21
	2,3-dimethylpentane	5.20	8.27	1.67	2.62
	cyclohexane	5.52	18.73	1.90	6.23
	3-methyl hexane	11.79	14.71	3.77	4.87
	2,2,4-trimethyl pentane	15.49	11.97	7.11	6.11
	heptane	16.71	28.75	6.68	9.76
	methyl cyclohexane	11.59	13.46	4.40	5.18
	2, 3, 4-trimethylpentane	6.99	12.60	4.45	5.60
	2-methyl heptane	8.02	10.97	2.15	4.26
	3-methyl heptane	4.83	7.49	2.06	2.86
	n-octane	8.97	12.91	2.17	5.20
	n-nonane	3.89	4.25	0.58	2.13
	decane	1.34	2.44	0.18	1.72
	undecane	1.02	0.88	0.21	0.76
	dodecane	1.42	0.60	0.29	0.55
Alkene	ethylene	18.14	8.98	5.98	1.57
	propylene	21.34	18.11	8.59	6.62
	trans-2-butene	5.80	6.21	0.93	0.48
	1-butene	26.17	22.17	8.65	4.86
	cis-2-butene	3.99	4.92	0.89	0.45
	1,3-Butadiene	2.06	0.21	0.49	0.04
	1-pentene	8.32	10.55	2.18	2.23
	trans-2-pentene	9.78	4.90	1.23	1.15
	isoprene	2.34	0.22	0.42	0.06
	cis-2-pentene	6.24	2.92	0.75	0.67
	1-hexene	10.50	17.69	2.62	4.65
Aromatic hydrocarbon	benzene	28.69	17.64	15.40	7.34
	toluene	32.03	18.06	15.54	9.35
	ethyl benzene	4.11	8.57	2.27	4.07
	p-/m-xylene	10.36	7.04	5.54	4.66
	2-dimethyl benzene	3.79	10.07	1.94	4.96
	styrene	1.48	0.41	2.09	0.22
	cumene	0.40	0.44	0.10	0.21
	propylbenzene	0.34	0.83	0.11	0.48
	3-Ethyltoluene	0.68	2.36	0.29	1.38
	4-methylethylbenzene	0.50	1.01	0.16	0.61
	1,3,5-trimethyl benzene	0.61	1.29	0.19	0.82
	2-Ethyltoluene	0.36	1.05	0.11	0.66
	1,2,4-trimethyl benzene	0.91	1.29	0.32	0.82
	1,2,3-trimethyl benzene	0.44	0.90	0.10	0.67
	1,3-diethyl benzene	0.27	0.16	0.07	0.13
	1,4-diethyl benzene	0.49	0.67	0.13	0.56
	naphthalene	1.55	0.35	0.32	0.34
Other organics	dichloromethane	1.56	2.05	0.97	0.14
	methyl tertiary-butyl ether	0.23	1.36	0.13	0.61
	ethyl acetate	0.63	0.14	0.27	0.07
	2-butanone	11.47	6.97	6.53	1.87
	methyl methacrylate	0.05	8.79	0.03	3.32
	vinyl trichloride	2.81	3.19	2.44	1.36
	acrolein	24.90	13.43	8.64	5.20
	ethyl chloride	0.21	0.02	0.13	0.01
	acetone	69.74	28.27	30.37	8.06
	vinyl acetate	0.29	0.29	0.07	0.09
	chloroform	9.96	0.50	2.47	0.35
	tetrahydrofuran	0.57	0.19	0.10	0.07
	1,2-dichloroethane	1.29	0.20	0.59	0.12
	4-methyl-2-pentanone	1.08	0.36	0.38	0.19
	trichloroethylene	2.81	2.16	2.44	1.72
The total emission concentration of VOCs		720.4	694.3	236.5	199.6

## Data Availability

Data will be made available on request.

## References

[1] Lv Y., Wu S., Li N., Liu Q., Yang C., Zou Y., Amirkhanian S. (2024). Flue Gas Suppression and Environmental Evaluation of Deodorizer-Modified Rubber Asphalt Based on Radar Method. Constr. Build. Mater..

[2] Radziszewski P., Liphardt A., Sarnowski M., Kowalski K.J., Pokorski P., Konieczna K., Król J.B., Iwański M., Chomicz-Kowalska A., Maciejewski K. (2023). Ageing evaluation of foamed polymer modified bitumen with bio-flux additive. Materials.

[3] Vidal R., Moliner E., Martínez G., Rubio M.C. (2013). Life Cycle Assessment of Hot Mix Asphalt and Zeolite-Based Warm Mix Asphalt with Reclaimed Asphalt Pavement. Resour. Conserv. Recycl..

[4] Yang X., Wang G., Rong H., Meng Y., Liu X., Liu Y., Peng C. (2022). Review of Fume-Generation Mechanism, Test Methods, and Fume Suppressants of Asphalt Materials. J. Clean. Prod..

[5] Espinoza J., Medina C., Calabi-Floody A., Sánchez-Alonso E., Valdés G., Quiroz A. (2020). Evaluation of Reductions in Fume Emissions (VOCs and SVOCs) from Warm Mix Asphalt Incorporating Natural Zeolite and Reclaimed Asphalt Pavement for Sustainable Pavements. Sustainability.

[6] Xu H., Zou Y., Gordon A., Wang H., Zhang H., Wu S., Chen A. (2024). Wetting of biorejuvenator nanodroplets on bitumen: A molecular dynamics investigation. J. Clean. Prod..

[7] Valdés-Vidal G., Calabi-Floody A., Sanchez-Alonso E., Díaz C., Fonseca C. (2020). Highway Trial Sections: Performance Evaluation of Warm Mix Asphalt and Recycled Warm Mix Asphalt. Constr. Build. Mater..

[8] Wang H., Liu Q., Wu S., Lv Y., Wan P., Gong X., Liu G. (2023). Study of Synergistic Effect of Diatomite and Modified Attapulgite on Reducing Asphalt Volatile Organic Compounds Emission. Constr. Build. Mater..

[9] Arabani M., Pirbasti Z.R., Hamedi G.H. (2021). Investigating the Impact of Zeolite on Reducing the Effects of Changes in Runoff Acidity and the Moisture Sensitivity of Asphalt Mixtures. Constr. Build. Mater..

[10] Cui P., Schito G., Cui Q. (2020). VOC Emissions from Asphalt Pavement and Health Risks to Construction Workers. J. Clean. Prod..

[11] Yang C., Wu S., Cui P., Amirkhanian S., Zhao Z., Wang F., Zhang L., Wei M., Zhou X., Xie J. (2022). Performance characterization and enhancement mechanism of recycled asphalt mixtures involving high RAP content and steel slag. J. Clean. Prod..

[12] Cao L., Yang C., Li A., Wang P., Zhang Y., Dong Z. (2021). Flue Gas Composition of Waste Rubber Modified Asphalt (WRMA) and Effect of Deodorants on Hazardous Constituents and WRMA. J. Hazard. Mater..

[13] Zadshir M., Oldham D.J., Hosseinnezhad S., Fini E.H. (2018). Investigating Bio-Rejuvenation Mechanisms in Asphalt Binder via Laboratory Experiments and Molecular Dynamics Simulation. Constr. Build. Mater..

[14] Martinez-Soto A., Calabi-Floody A., Valdes-Vidal G., Hucke A., Martinez-Toledo C. (2023). Life cycle assessment of natural zeolite-based warm mix asphalt and reclaimed asphalt pavement. Sustainability.

[15] Woszuk A., Wróbel M., Franus W. (2019). Application of Zeolite Tuffs as Mineral Filler in Warm Mix Asphalt. Materials.

[16] Wang M., Wang C., Huang S., Yuan H. (2021). Study on Asphalt Volatile Organic Compounds Emission Reduction: A State-of-the-Art Review. J. Clean. Prod..

[17] Wang S., Yang C., Zhao J., Li C., Fan X. (2023). Rapid and Direct Assessment of Asphalt Volatile Organic Compound Emission Based on Carbon Fiber Ionization Mass Spectrometry. ACS Omega.

[18] Hasan M., Sugiarto S. (2021). Determining the Properties of Semi-Flexible Pavement Using Waste Tire Rubber Powder and Natural Zeolite. Constr. Build. Mater..

[19] Wang C., Wang M., Chen Q., Zhang L. (2022). Basic Performance and Asphalt Smoke Absorption Effect of Environment-Friendly Asphalt to Improve Pavement Construction Environment. J. Clean. Prod..

[20] Li Z., Ren J., Zhu J., Li W., Fu X., Yang L. (2020). Study on the Construction Performance of Zeolite Asphalt Mixture Based on Macro-Micro Scale edited by M. Guo. Adv. Mater. Sci. Eng..

[21] Wang M., Li P., Nian T., Mao Y. (2021). An Overview of Studies on the Hazards, Component Analysis and Suppression of Fumes in Asphalt and Asphalt Mixtures. Constr. Build. Mater..

[22] Al-Hadidy A.I., Alzeebaree R., Abdal J.A., Niş A. (2023). Mechanical Performance and Statistical Analysis of Natural and Synthetic Zeolite-Warm Mix Asphalt as a Function of Compaction Efforts. J. Build. Eng..

[23] Han X., Yu J., Cao Z., Wang R., Du W., He P., Ge Y. (2020). Preparation and Properties of Silane Coupling Agent Modified Zeolite as Warm Mix Additive. Constr. Build. Mater..

[24] Ikhlaq A., Kasprzyk-Hordern B. (2017). Catalytic Ozonation of Chlorinated VOCs on ZSM-5 Zeolites and Alumina: Formation of Chlorides. Appl. Catal. B Environ..

[25] Li N., Jiang Q., Wang F., Xie J., Li Y., Li J., Wu S. (2020). Emission Behavior, Environmental Impact and Priority-Controlled Pollutants Assessment of Volatile Organic Compounds (VOCs) during Asphalt Pavement Construction Based on Laboratory Experiment. J. Hazard. Mater..

[26] Chang X., Long Y., Wang C., Xiao Y. (2023). Chemical Fingerprinting of Volatile Organic Compounds from Asphalt Binder for Quantitative Detection. Constr. Build. Mater..

[27] Liu N., Liu L., Li M., Sun L. (2023). Effects of Zeolite on Rheological Properties of Asphalt Materials and Asphalt-Filler Interaction Ability. Constr. Build. Mater..

[28] Muscarella S.M., Badalucco L., Cano B., Laudicina V.A., Mannina G. (2021). Ammonium Adsorption, Desorption and Recovery by Acid and Alkaline Treated Zeolite. Bioresour. Technol..

[29] Sanchez-Alonso E., Valdes-Vidal G., Calabi-Floody A. (2020). Experimental Study to Design Warm Mix Asphalts and Recycled Warm Mix Asphalts Using Natural Zeolite as Additive for Sustainable Pavements. Sustainability.

[30] Sharma A., Lee B.-K. (2017). A Novel Nanocomposite of Ca(OH)_2_ -Incorporated Zeolite as an Additive to Reduce Atmospheric Emissions of PM and VOCs during Asphalt Production. Environ. Sci. Nano.

[31] Woszuk A., Zofka A., Bandura L., Franus W. (2017). Effect of Zeolite Properties on Asphalt Foaming. Constr. Build. Mater..

[32] Wu R., Xiao Y., Zhang P., Lin J., Cheng G., Chen Z., Yu R. (2022). Asphalt VOCs Reduction of Zeolite Synthesized from Solid Wastes of Red Mud and Steel Slag. J. Clean. Prod..

[33] Al-Saffar Z.H., Yaacob H., Katman H.Y., Mohd Satar M.K.I., Bilema M., Putra Jaya R., Eltwati A.S., Radeef H.R. (2021). A review on the durability of recycled asphalt mixtures embraced with rejuvenators. Sustainability.

[34] Cui P., Wu S., Xiao Y., Hu R., Yang T. (2021). Environmental performance and functional analysis of chip seals with recycled basic oxygen furnace slag as aggregate. J. Hazard. Mater..

[35] Xiao Y., Chang X., Yan B., Zhang X., Yunusa M., Yu R., Chen Z. (2021). SBS Morphology Characteristics in Asphalt Binder and Their Relation with Viscoelastic Properties. Constr. Build. Mater..

[36] Zeng S., Mao S., Xu S., He Y., Yu J. (2024). Investigation on DOPO as Reactive Fumes Suppressant to Reduce the Fumes Emission of Asphalt. J. Hazard. Mater..

[37] Zou F., Leng Z., Cao R., Li G., Zhang Y., Sreeram A. (2022). Performance of Zeolite Synthesized from Sewage Sludge Ash as a Warm Mix Asphalt Additive. Resour. Conserv. Recycl..

[38] Zhang X., Xiao Y., Long Y., Chen Z., Cui P., Wu R., Chang X. (2021). VOCs Reduction in Bitumen Binder with Optimally Designed Ca(OH)_2_-Incorporated Zeolite. Constr. Build. Mater..

[39] Zhang T. (2020). Atmospheric Diffusion Profiles and Health Risks of Typical VOC: Numerical Modelling Study. J. Clean. Prod..

[40] Lv Y., Wu S., Li N., Cui P., Wang H., Amirkhanian S., Zhao Z. (2023). Performance and VOCs Emission Inhibition of Environmentally Friendly Rubber Modified Asphalt with UiO-66 MOFs. J. Clean. Prod..

